# Valorising Cassava Peel Waste Into Plasticized Polyhydroxyalkanoates Blended with Polycaprolactone with Controllable Thermal and Mechanical Properties

**DOI:** 10.1007/s10924-023-03167-4

**Published:** 2024-01-27

**Authors:** Emma Martinaud, Carmen Hierro-Iglesias, James Hammerton, Bawan Hadad, Rob Evans, Jakub Sacharczuk, Daniel Lester, Matthew J. Derry, Paul D. Topham, Alfred Fernandez-Castane

**Affiliations:** 1 École Nationale Supérieure de Chimie, de Biologie et de Physique, Polytechnic Institute of Bordeaux, 33607 Pessac Cedex, France; 2 Energy and Bioproducts Research Institute, Aston University, Birmingham, B4 7ET UK; 3 Aston Advanced Materials Research Centre, Aston University, Birmingham, B4 7ET UK; 4 Polymer Characterisation Research Technology Platform, University of Warwick, Coventry, CV4 7AL UK

**Keywords:** Renewable feedstock, Waste valorisation, Biotransformation, Biopolymer blends, Tuneable properties

## Abstract

Approximately 99% of plastics produced worldwide were produced by the petrochemical industry in 2019 and it is predicted that plastic consumption may double between 2023 and 2050. The use of biodegradable bioplastics represents an alternative solution to petroleum-based plastics. However, the production cost of biopolymers hinders their real-world use. The use of waste biomass as a primary carbon source for biopolymers may enable a cost-effective production of bioplastics whilst providing a solution to waste management towards a carbon–neutral and circular plastics economy. Here, we report for the first time the production of poly(hydroxybutyrate-*co*-hydroxyvalerate) (PHBV) with a controlled molar ratio of 2:1 3-hydroxybutyrate:3-hydroxvalerate (3HB:3HV) through an integrated pre-treatment and fermentation process followed by alkaline digestion of cassava peel waste, a renewable low-cost substrate, through *Cupriavidus necator* biotransformation. PHBV was subsequently melt blended with a biodegradable polymer, polycaprolactone (PCL), whereby the 30:70 (mol%) PHBV:PCL blend exhibited an excellent balance of mechanical properties and higher degradation temperatures than PHBV alone, thus providing enhanced stability and controllable properties. This work represents a potential environmental solution to waste management that can benefit cassava processing industries (or other crop processing industries) whilst developing new bioplastic materials that can be applied, for example, to packaging and biomedical engineering.

**Graphical Abstract:**

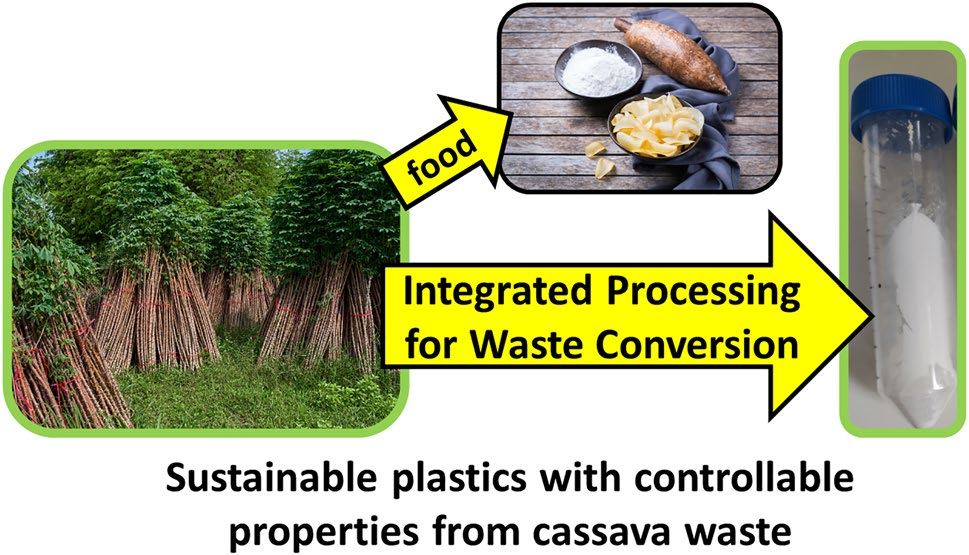

**Supplementary Information:**

The online version contains supplementary material available at 10.1007/s10924-023-03167-4.

## Introduction

In 2019, 99% of plastics produced worldwide were produced by the petrochemical industry [1]. In the UK, an estimated 4.9 million metric tons of plastics were placed on the market in 2018, of which three-quarters became waste [2]. It is expected that plastic waste will increase by 20% by 2030 due to the increase in plastic demand [3]. This represents a crucial need to promote the use of sustainable plastics of biological origin (so-called ‘bioplastics’ or ‘biopolymers’) instead of petroleum-based plastics that do not degrade, which are causing excessive pollution in the environment.

Biopolymers can be used in a wide range of applications such as commodity plastics for packaging and agricultural use, and medical applications [4–8]. However, the high cost of biopolymer production is closely associated with the cost of raw materials (i.e. carbon source and associated extraction/conversion) and this hinders their deployment for commercial use [9]. Among the many biopolymers, polyhydroxyalkanoates (PHAs) are linear aliphatic polyesters synthesized by microorganisms and accumulated as aggregates in the cytoplasm of prokaryotic cells [10]. PHAs offer many environmental advantages over petroleum-based plastics. For example, recent studies report ca. 80% reduction in global warming potential for the production of 1 kg of PHAs compared to petrochemical alternatives such as polypropylene (PP) or polyethylene (PE) [8]. In addition, the biological production of polymers circumvents the limitations of conventional polymer synthesis cutting out the need for monomer isolation and chemical polymerisation methods [11].

The use of renewable resources, such as waste biomass, as a primary carbon source for biopolymers opens up new avenues to decrease production costs of bioplastics whilst providing an effective solution to waste management towards a carbon–neutral and circular plastics economy. Activities from the agricultural sector and crop processing industries result in the generation of large quantities of waste, presenting major management and environmental issues. However, this can be seen as an opportunity for the waste to be used as the feedstock in PHA production. Sugarcane, rice, cassava, palm oil and jatropha are some of the most relevant crops worldwide from which large quantities of waste are generated [12]. PHAs has been previously produced from waste such as lignocellulosic biomass [13], sugarcane molasses [14], and wastewater [15]. Relevant to this work, cassava (*Manhiot esculenta*) is an important source of farm income in Sub-Saharan Africa (SSA) [16]. Around 169 Mt of cassava are produced in Africa annually [17] resulting in 40 Mt of cassava waste [18], and each ton of cassava pulp abandoned in landfills releases between 195 and 361 kg of CO_2_ equivalent to the atmosphere [19]. Cassava waste includes peels, bagasse and wastewater; which have the potential for valorisation into products (i.e. starch, bioethanol and biofuel) [20]. Previous studies have demonstrated the production of PHA from cassava starch [21, 22] and cassava waste [23]. However, the production of PHA from cassava waste is scarce and a recent review addressed the numerous opportunities for the development of cassava waste biorefineries to produce PHA in SSA [24].

Because of the differing properties, PHA can be categorised by the macromolecular chain length; short-chain length PHA (scl-PHA) with molecular chains of fewer than five backbone carbon atoms, medium-chain length PHA (mcl-PHA) whereby the molecular chains comprise six to fourteen backbone carbon atoms and long-chain length PHA (lcl-PHA) with molecular chains longer than fourteen backbone carbon atoms [25]. The two most common PHA types of biological origin are polyhydroxybutyrate (PHB) and poly(hydroxybutyrate-*co*-hydroxyvalerate) (PHBV), both scl-PHA thermoplastic biopolymers with low glass-transition temperature (*T*_g_) of 3–8 ℃ and high melting points (*T*_m_) of 160–180 ℃ [26]. The thermal and mechanical properties of the PHA are largely influenced by the pendant group on their chain*,* whereas the backbone chain ester linkages result in PHA being biodegradable polymers and these properties make them attractive for a wide range of applications such as packaging [27] or as drug carriers [28]. In biomedical engineering, PHA are useful for various purposes. For example, surgical attire, sutures, and vascular implants can be made using PHA [29]. The major limitation of PHA alone is their inferior mechanical properties compared to more conventional plastics (e.g. PE) and effort should be focused on improving their flexibility and strength in order to fully exploit the potential of these biopolymers. Many biologically derived polymers (e.g. softwood kraft lignin [30]) have disadvantages for general use, such as being brittle with a high Young’s modulus and short elongation, and PHA is no exception. The co-polymerization of 3-hydroxybutyrate (HB) and 3-hydroxyvalerate (3HV) in varying proportions yields poly(3-hydroxybutyrate-*co*-3-hydroxyvalerate) (PHBV) which has higher elongation than PHB [31]. Increasing the molar ratio (mol%) of 3HV in PHBV enhances the polymer elongation but reduces the melting temperature, glass transition temperature, and tensile strength [32]. Thus, alteration of the 3HV mol% in PHBV polymer helps to diversify its material properties, concomitantly broadening its practical use. On the other hand, a relatively simple cost-effective approach to enhance the mechanical properties of a (bio)polymer is to blend it with other (bio)polymers, such as starch, poly(lactic acid) (PLA) or poly(butylene succinate) (PBS) [33–38]. PLA/PHA blends have been created using melt blending, as the thermal properties of these two polymers are quite similar. As an example, this blending improved the elongation at break from 4 to 200%, when just a small amount of PHA was mixed with PLA [33]. For a comprehensive review of biopolymer blends, the reader is directed elsewhere [39].

**Scheme 1:**
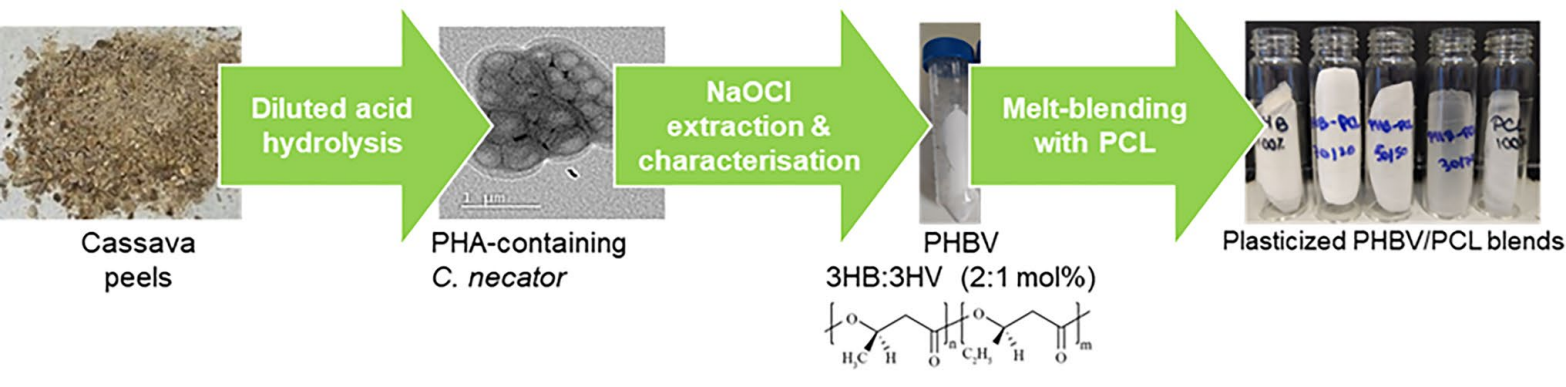
Overall process converting cassava peels into PHA/PCL bioplastic blends

Polycaprolactone (PCL) is a semicrystalline aliphatic polyester, with high crystallinity and flexibility, used extensively in biomedical applications [40, 41]. PCL intermediary products, such as adipic acid, can be biosynthesized from renewable resources although industrially, PCL is predominantly synthesized from petroleum-derived chemicals [42]. PCL has been used for various purposes in tissue engineering such as for the production of scaffolds for the repair of cartilage and bone [43]. There are currently only a few reports on PHBV/PCL blends [44–46] and the vast majority of such studies decouple the production of PHA from renewable sources (*i.e.* waste biomass) from the blending of PHA (often using commercially sourced PHA) with other biopolymers to synthesize plastic materials. Our work begins to fill this gap by employing cassava peel waste (as a huge agricultural waste resource) to extract fermentable sugars for microbiological transformation into the co-polymer PHBV using *Cupravidus necator*. We have recently developed an integrated process for the conversion of cassava peel waste into PHA via diluted acid pre-treatment followed by fermentation of cassava peel hydrolysate (CPH) as a sole carbon source to produce PHBV [47]. Building on the pioneering work developed in our laboratories, in this work, PHBV has been produced from cassava peel hydrolysate, extracted and characterized. Subsequently, PHBV was blended with PCL to produce plasticized materials with improved thermal and mechanical properties toward developing sustainable materials for biomedical applications (Scheme 1).

## Materials and Methods

### Pre-treatment of Cassava Peels

Cassava peels were sampled from a small cassava processing plant in Bawjiase (Ghana) and were kindly donated by Dr Bayitse (CSIR, Ghana). Mechanical pre-treatment and particle size reduction were carried out as described elsewhere [48]. Briefly, cassava peels were soaked in water for 30 min to ease the removal of the brown skin, then dried at 60 ℃ overnight and finally milled. The resulting dried cassava powder showed a total carbohydrates composition of 88% (dry matter) determined according to the protocol A0003 from Enzyme Lab of DTI (Denmark).

Diluted acid hydrolysis of cassava peels was performed as described by Hierro-Iglesias and co-workers [47]. Briefly, hydrolysis was carried out using a Starfish workstation (Radleys, Essex, UK) equipped with a heating plate to regulate temperature. 20 mL reaction volume containing 10% (w/v) of solids in 0.6 M H_2_SO_4_ were incubated for 58 min at 107 ºC using 50 mL two-neck round bottom pressure flasks (Aldrich®, Merck, Darmstadt, Germany). Subsequently, the cassava peel hydrolysate (CPH) was filtered to remove the fibres via vacuum filtration through a cellulose 11 µm filter paper (Whatman, Cytiva, Washington D.C, United States) and neutralized to pH 7.00 using 12.5 M NaOH solution.

### Strain, Media and Culture Conditions

A *C. necator* H16-derived strain capable of metabolising glucose was obtained by adaptative evolution and used in this study. The strain was kindly provided by Dr Kovacs (University of Nottingham, United Kingdom) and the mutation protocol was adapted from the methodology established by Franz and coworkers [49]. Cryostocks were prepared in 15% (w/v) glycerol and stored at − 80 ℃ until used. Pre-inoculum culture media containing (g/L): 10, peptone; 10, beef extract; 5, NaCl was prepared, adjusted to pH 7 with NaOH and sterilised at 121 ℃ for 20 min in an autoclave (Priorclave, Ltd., London, UK). Seed culture media composition was as follows (g/L): 10 glucose; 1 (NH_4_)_2_SO_4_; 1.5 KH_2_PO_4_; 9 Na_2_HPO_4_ 2H_2_O; 0.2 MgSO_4_ 7H_2_O; 9 citric acid and 1 ml/L trace element solution. The pH of the medium was adjusted to 7.00 with NaOH and subsequently sterilised by autoclaving at 121 ℃ for 20 min. Glucose and phosphates were autoclaved separately and added aseptically after autoclaving. Trace elements solution was added after autoclaving and sterilised by filtration through a 0.22 µm cellulose filter (Sartorius Stedim UK Ltd, Surrey, UK). Trace elements solution was composed of (g/L): 10 FeSO_4_ · 7H_2_O; 2.25 ZnSO_4_ · 7H_2_O; 1 CuSO_4_ · 5H_2_O; 0.5 MnSO_4_ · 5H_2_O; 2 CaCl_2_ · 2H_2_O; 0.23 Na_2_B_4_O7 · 10H_2_O; 0.1 (NH_4_)_6_Mo_7_O_24_ and 10 ml/L 35% HCl [50]. Batch media used in bioreactor cultures consisted of CPH diluted to a glucose concentration of 20 g/L. Yeast extract at a final concentration of 1 g/L was added at the end of the exponential phase.

For all experiments, 1 mL of cryostock was inoculated in 10 mL of pre-inoculum media and incubated overnight at 30 ºC and 250 rpm in an orbital shaker incubator (Incu-Shake MAXI®, SciQuip Ltd, Newtown, UK). Seed cultures used for bioreactor inoculation were inoculated to achieve an initial OD_600_ of 0.2 using pre-inoculum cultures and were incubated for 8 h at 30 ℃ and 250 rpm in an orbital shaker incubator (Incu-Shake MAXI®, SciQuip Ltd, Newtown, UK). The bioreactor was inoculated to achieve an initial OD_600_ of 0.2 using a seed culture to a final volume of 700 mL. Biostat B (Sartorius Stedim UK Ltd, Surrey, UK) automated laboratory 1 L jacketed vessels equipped with four baffles and an agitator with 2 six-bladed Rushton turbines were used. Dissolved oxygen in the medium (pO_2_) was measured online using an OxyFerm FDA VP 160 probe (Hamilton, Bonaduz, Switzerland) and maintained above 20% using a cascade control for aeration. Aeration was achieved by sparging air from below the lower impeller at 1 vvm, through a 0.22 µm filter (Sartorius Stedim UK Ltd, Surrey, UK). Agitation was maintained between 300 and 500 rpm. pH was monitored online using an Easyferm plus PHI VP 160 Pt100 Probe (Hamilton, Bonaduz, Switzerland) and maintained at 7.00 ± 0.05 using 1 M HCl and 2 M NaOH. The broth was harvested at the end of the exponential phase and subsequently centrifuged at 4,000 rpm for 15 min using a Sorvall™ Lynx 4000 centrifuge (Thermo Scientific™, Waltham, USA). The supernatant was discarded and the pellet was stored at − 80 ℃ until further use.

### Extraction of PHBV by Alkaline Digestion

The bacterial pellet was freeze-dried overnight in a benchtop freeze-dryer (Lablyo, Frozen in Time Ltd, York, UK) at − 50 ℃ and 1–5 Pa. Following freeze-drying, 400 mg of pulverized cells (30 g L^−1^) were suspended in 20 mL aqueous 13% (w/v) sodium hypochlorite solution (NaClO) at pH 12.3 and incubated at room temperature for 1 h. Then, distilled H_2_O was added to make up a 50% increase in volume to enhance the PHBV sedimentation rate, incubated at room temperature, and left to stand for 16 h. The upper phase (containing water-soluble components) was discarded. The bottom phase (containing PHBV) was washed twice by centrifugation (Thermo Scientific Heraeus Multifuge X1R) for 10 min at 4000 g_av_ at 4 ℃ with an equal volume of distilled H_2_O and resuspended with 2 mL of isopropanol. Subsequently, the solution was freeze-dried overnight (Lablyo, Frozen in Time Ltd, York, UK), weighed using an analytical balance (ABJ-NM, KERN & SOHN GmbH, Balingen, Germany), and stored at room temperature in a desiccator with silica gel beads until further analysis.

### Solvent Blending of Binary Films

The composition of PHBV/PCL binary blends for solvent blending was selected with ratios of 70/30, 50/50, and 30/70 (% w/w). 150 mg of total biopolymers were mixed in 10 mL chloroform at 60 ℃ continuously stirring at 160 rpm until dissolved. Subsequently, the solution was cast into a PTFE beaker under a fume hood at room temperature for at least 2 days to allow complete evaporation of the solvent. For comparison, 100% PHBV and 100% PCL were processed under the same conditions.

### Analytical Techniques

#### Determination of Microbial Growth

Microbial growth was analysed by optical density at a wavelength of 600 nm (OD_600_) using a Jenway 6310 Spectrophotometer (Keison Products, Chelmsford, UK).

#### Analysis of Glucose Concentration

Glucose concentration was determined in CPH and fermentation samples as follows: 1 mL samples were filtered using PVDF 0.45 µm filters (Whatman, Cytiva, Washington D.C, United States) and concentration was measured using a YSI biochemical analyser (model 2500, Xylem Inc., Nottingham, UK).

#### Polyhydroxyalkanoates Imaging Using Fluorescence Microscopy

*C. necator* cells (100 μL) were taken from the bioreactor culture and stained with 5 μL of 0.1 mg mL^−1^ of pyrromethene-546 (Pyr-546) in dimethyl sulfoxide for polyhydroxyalkanoates (PHA) imaging [51]. A Zeiss Primo Star iLed microscope (Carl Zeiss Ltd., Cambridge, UK) fitted with a Zeiss AxioCam ERc 5 s camera was used. Images were acquired within 1 min of fluorophore incubation and processed with the aid of Zeiss ZEN Lite 2012 software in auto exposure mode. Samples were excited with a Zeiss Led 470 nm light source and a 515 LP filter was employed for detection of Pyr-546 fluorescence.

#### Quantification of PHA Production Using Gas Chromatography-Mass Spectrometry

PHA quantification was performed by acid propanolysis followed by GC–MS analysis. Propanolysis was performed using 5 mg of lyophilized bacteria in 1 mL of chloroform and 1 mL of 1-propanol containing 15% of 37% (v/v) HCl in an 8 mL glass tube (Duran®, DWK Life Sciences, Mainz, Germany). 50 µL of 1 mg/mL solution of benzoic acid in 1-propanol was added as an internal standard. The mixtures were incubated for 2 h at 100 ℃ in an oven (Memmert, Schwabach, Germany). After cooling to room temperature, 1 mL of distilled water was added followed by vortexing for 30 s before being left to stand for 5 min to allow for phase separation. The upper aqueous phase was discarded, and the washing step was repeated to eliminate impurities. 1 mL of the organic phase containing the resulting PHA was filtered with 0.2 µm nylon filters (Fisher Scientific, Loughborough, UK) and transferred to a GC–MS vial. The standard sample of poly(3-hydroxybutyric acid-*co*-3-hydroxyvaleric acid) (80181-31-3, Merck, Darmstadt, Germany) was used to prepare the standard calibration curve. Analysis was performed using a single quadrupole gas chromatograph-mass spectrometer GCMS-QP2010 (Shimazu, Milton Keynes, UK). Injection of 1 µL samples was carried to an analyser with a 30 m SH-Rtx-5MS column (Shimazu, Milton Keynes, UK) at 280 ℃. The flow rate was set at 1.25 mL/min and the ionization detector at 240 ℃. The column temperature was programmed from 40 to 250 ℃ at a rate of 5 ℃/min. The total measurement time for each sample was 40 min. PHBV was identified using the NIST17s and NIST17-1 internal libraries available in the GCMS Postrun Analysis Software (Shimazu, Milton Keynes, UK). The PHB and PHBV contents of each sample was normalised by the weight of the lyophilised bacteria and expressed as a percentage of polymer weight/ cell dry weight.

The PHA recovery yield was calculated as follows:

1 $$Yield \left(\%\right)= \frac{Mass of freeze-dried extract }{Mass of cells used for extraction \times PHA in cells \left(\%\right)} \times 100$$

#### Thermogravimetric Analysis

Thermogravimetric analysis (TGA) was conducted to test the thermal stability of PHBV after alkaline extraction. The analysis was conducted using a TGA analyzer (TGA 500Q, TA instruments, Etten-Leur, The Netherlands) in an inert atmosphere with N_2_. Approximately 5 mg of sample were placed in platinum crucibles and heated to 500 ℃ at a heating rate of 10 ℃/min. The mass of extracted PHA in each sample was determined as the mass loss in the temperature range between 232 and 274 ℃. The mass of commercial poly(3-hydroxybutyric acid-*co*-3-hydroxyvaleric acid) (PHBV) was determined as the mass loss in the temperature range between 290 and 325 ℃.

The purity of PHA was calculated as follows:

2 $${Purity}_{TGA} (\%)= \frac{Mass of PHA }{Total mass of sample}\times 100$$

#### Differential Scanning Calorimetry

The thermal properties of PHA and binary blends were investigated using Differential Scanning Calorimetry (DSC, Perkin Elmer Pyris I). 2–4 mg of sample were placed in aluminium crucibles and were first heated from 50 to 200 ℃ at a heating rate of 10 ℃/min, then cooled from 200 to − 100 ℃ at a cooling rate of 5 ℃/min. A second heating cycle was conducted from − 100 to 200 ℃ at a heating rate of 5 ℃/min under a nitrogen atmosphere.

#### Fourier Transform Infrared Spectroscopy

Attenuated total reflection Fourier transform infrared (ATR-FTIR) spectroscopy was carried out with a PerkinElmer Spectrum One, Hopkinton, MA, USA (400–4000 cm^−1^) equipped with a ZnSe crystal.

#### Gel Permeation Chromatography

Gel permeation chromatography (GPC) was used to study the molecular weight of extracted PHA and compared with commercial PHBV. GPC analysis was carried out using an Agilent 1260 Infinity II instrument with a refractive index (RI) detector equipped with PLGel Mixed C 7.8 × 300 mm column (molecular weight resolving range = 2000–4,000,000 g/mol) at 30 ℃ and calibrated with PMMA (Agilent EasiVials). Biopolymers (5 mg) were dissolved with distilled chloroform (99,8% (w/v), Sigma Aldrich) which was used as the mobile phase.

#### ^1^H Nuclear Magnetic Resonance spectroscopy

Proton Nuclear Magnetic Resonance (^1^H-NMR) spectroscopy was carried out to study the chemical structures of the extracted PHA using a Bruker-300 Ultra Shield, 300 MHz. Biopolymers (3 mg) were dissolved in 5 mL of chloroform-D (CDCl_3_). Chemical shifts were referenced to the CHCl_3_ residual solvent peak at δ = 7.26 ppm.

#### X-Ray Diffraction

The crystallinity of blended film samples was investigated by X-ray diffraction (XRD, Rigaku X-ray diffractometer), operating at 40 kV and 20 mA with a diffraction angle range (2θ) from 3 to 60 degrees at a scan rate of 4°/min.

#### Mechanical Properties

The mechanical properties of the blended films were observed using a universal tensile testing machine (INSTRON® CALIBRATION LAB, Model 5965, Hopkinton, MA, USA), according to the ASTM D638 standard test for tensile properties of plastics. The blended film samples were cut to 10 × 4 × 2 (LxWxD) mm. A load cell of 50 N and an extension rate of 5 mm/min were employed and stress–strain curves were then analysed.

## Results and Discussion

### PHA Production

In our recent work, we have developed an integrated process for the conversion of cassava peels into PHA via an acid pre-treatment followed by fermentation of the cassava peel hydrolysate (CPH) as a sole carbon source to produce PHA in bioreactor cultures [47]. Building on the pioneering work developed in our previous work, a batch culture was carried out reaching a biomass growth of 3.4 g dry cell weight per liter (DCW/L), which corresponds to OD_600_ 12.1 in 59 h and GC–MS analysis revealed a PHA content of 28.6% (g PHA/100 g DCW) in mass, equivalent to 1 g/L of PHA. Figure S1 and Table S1 shows the fermentation profiles and the derived bioprocess parameters, respectively. To the best of our knowledge, prior to our investigation, scarce studies have been published on the biotransformation of cassava waste into PHA by *C. necator.* Poomipuk and coworkers achieved a PHA production of 2.4 g/L, equivalent to 61.6% (g PHA/100 g DCW) [22]. While this outcome represents a twofold increase in PHA production, several significant disparities exist between their experimental approach and ours. In our research, we utilised cassava peels as substrate, in contrast to the previous study use of cassava starch. Furthermore, they enriched the media with all the essential nutrients to support *C. necator* growth, while our study exclusively used CPH to minimise process costs. Finally, different scales were used, while the authors performed the cultures at flask-scale, we carried out a fed-batch culture using a stirred tank bioreactor, an experimental strategy that resembles industrial settings.

**Fig. 1:**
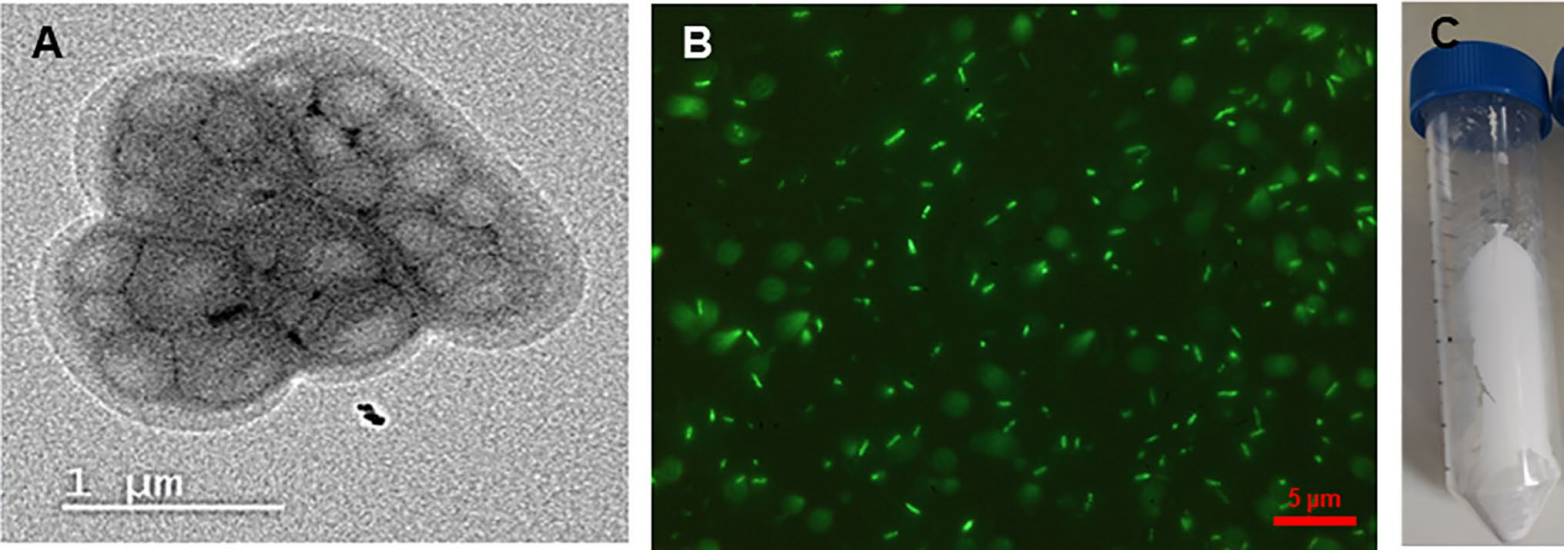
*Cupravidus necator* cells containing PHA granules produced from cassava peel hydrolysate: **A** TEM image and **B** Fluorescent microscopy image of Pyr-546-stained cells. **C** Photograph of the extracted PHA from *Cupravidus necator* using NaOCl

Figure 1 shows the accumulation of PHA granules in *C. necator* cells using TEM and fluorescent microscopy. The latter is an inexpensive and rapid method to qualitatively determine the presence of PHA in Pyr-546-stained cells [52]. PHA production can be as high as 90% of the bacterial dry cell weight under optimized conditions [53] and therefore, our results are below optimal accumulation. *C. necato*r produces PHA natively, mainly PHB, in the presence of an excess of carbon and under nutrient stress [54]. Although PHB is the most abundant biopolymer synthesized by *C. necator*, this bacterium can also produce copolymers such as poly(3-hydroxybutyrate-*co*-3-hydroxyvalerate) under appropriate conditions. Here we demonstrate that the use of CPH combined with our bioprocessing strategy, enables the production of PHBV. This copolymer has many advantages over other types of PHA such as toughness and elasticity [55]. The resulting biopolymer composition depends on different variables such as the carbon source or the biochemical pathway utilized [56]. While the most common approach for the synthesis of 3-HV units in PHBV is through the addition of precursors like propionic acid, prior research has documented the generation of fatty acids such as butyric and propionic acid through the acid hydrolysis of vegetable peels, including potatoes and onions [57]. Earlier studies have demonstrated the production of the PHBV copolymer from cassava peel hydrolysate without the need for adding exogenous precursors to the culture [58]. We indeed analysed by HPLC the presence of propionic, isovaleric, acetic, valeric, caproic and butyric acids in CPH. However, none of the acids were detected. We therefore hypothesise that the cultivation conditions utilised in this work in combination with the growth media composition and operational strategy, stimulate the synthesis of propionic acid due to the upregulation of the methyl citrate cycle of *C. necator* which may be subsequently utilised to form PHBV. We plan in future work to address this outstanding and intriguing research question.

### PHA Extraction and Characterisation

Cell digestion was carried out with NaOCl as described in the experimental section. Generally, sodium hypochlorite forms hypochlorous acid (HOCl) when it comes in contact with water. The cell membrane and exopolysaccharide (EPS) dissolve in HOCl, producing monochloramine and water. The large-scale application of this NaOCl method can be considered a safe method due to its non-combustible and non-volatile components [59]. Minimal hazards are associated with the concentrations of NaOCl used in this study (i.e., 13% (v/v)) and yet we were able to achieve a recovery yield of 97.2% (w/w) with a 93.5–94.0% (w/w) purity (Fig. 1C). For large scale NaOCl applications, i.e. > 1 m^3^, 10% (v/v) NaOCl with 3% (w/v) biomass loading was applied on a pure culture strain (*Ralstonia eutropha* H16) with a 91% PHA recovery yield [59]. Thus, our results are comparable to previous work and are scalable, an important factor for readers interested in techno-economic analysis and life cycle assessment.

**Fig. 2:**
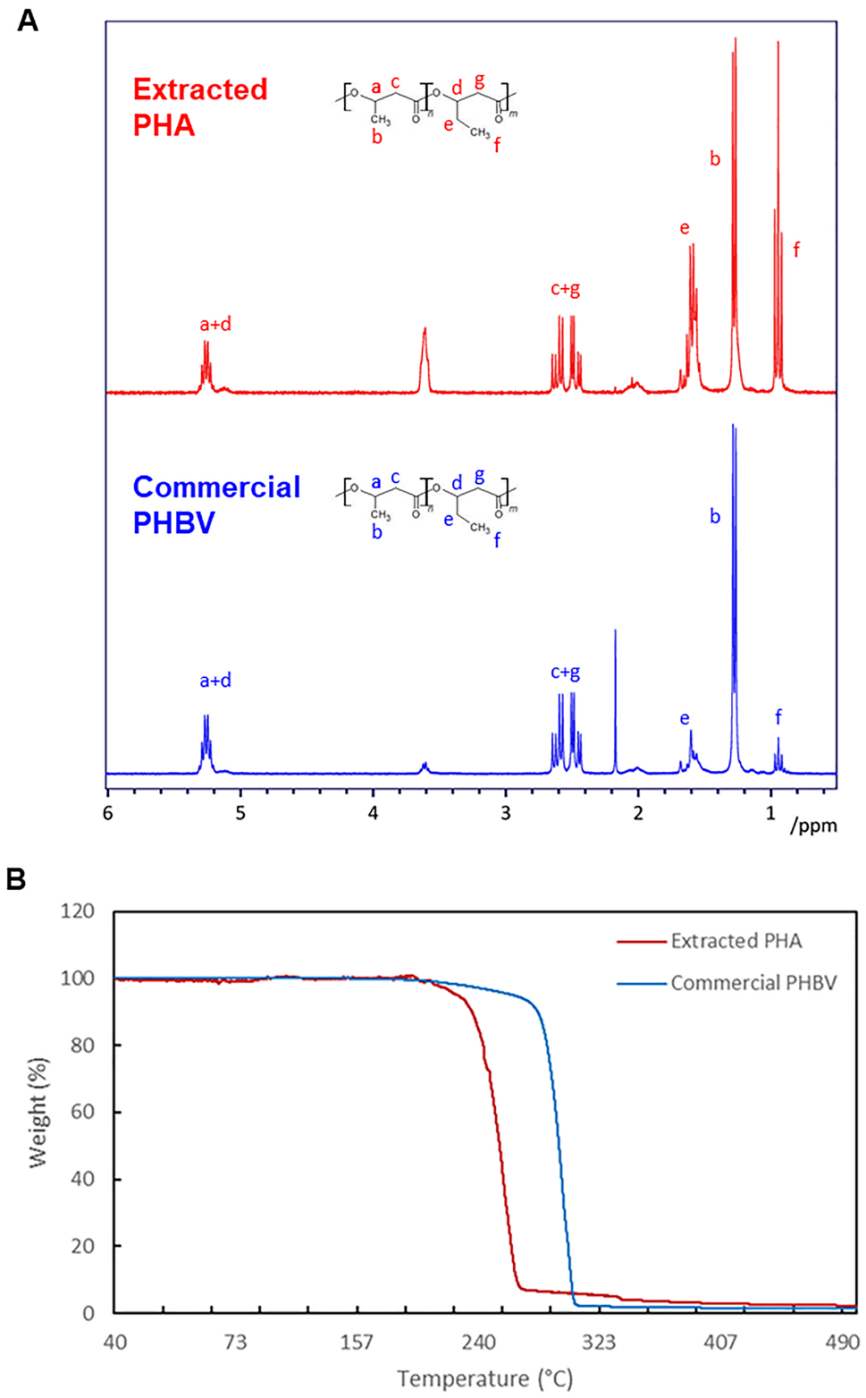
Characterisation of PHA samples. **A** ^1^H NMR spectra and **B** TGA thermograms of PHA extracted from *Cupravidus necator* cells grown on cassava peels hydrolysate, and commercial PHBV

^1^H NMR analysis was performed on the PHA samples extracted from *C. necator* cells grown on cassava peel hydrolysate and compared to commercial PHBV (Fig. 2A). The resonances at 0.88, 1.29, 1.7, 2.54, and 5.22–5.28 ppm correspond to –CH_3_ (HV), –CH_3_ (HB), –CH_3_ (HV), –CH_2_ (HV and HB), and –CH (HV and HB bulk structure), respectively. These signals indicate the presence of both PHB and PHV repeat units [60]. Commercial PHBV contains 92 mol% of 3HB and 8 mol% of 3HV. The copolymer composition for extracted PHA was calculated to be 64.5 mol% of 3HB and 35.5 mol% of 3HV, *i.e.* 3HB:3HV ratio of approximately 2:1, by comparing the methyl peaks in the ^1^H NMR spectrum corresponding to the 3HB and 3HV residues (peaks b and f in Fig. 2A, respectively). The non-annotated resonances at 4.2 ppm for the commercial PHB and at 3.6 ppm for our extracted PHA correspond to impurities revealing a 94% purity of the extracted PHA. Similarly, TGA analysis of extracted PHA (Fig. 2B) reveals a purity of 93.5% (w/w), in close agreement with NMR data. The presence of the PHBV copolymer was also confirmed by TGA as the extracted PHA showed a decomposition temperature (*T*_d_) of 245 ℃ which is in between the *T*_d_ of commercial PHB and PHBV (Table 1). These results provide evidence that the extracted PHA is a copolymer and not a blend.

**Table 1:** Composition molecular weight data, and thermal properties of PHA polymers

	HV:HB	Extraction of PHA		Molecular weights			Thermal properties	
Material	(%mol)	Recovery yield (%)	Purity (%)	*M*_n_ (g/mol)	*M*_*w*_ (g/mol)	*Đ* (*M*_w_/ *M*_n_)	T_d_ (°C)	T_m_ (°C)
Extracted PHA (this work)	35.5:64.5	97.2	93.5–94.0	606,378	933,284	1.54	245	167/173
PHBV (CAS 80181-31-3)	42.3:57.7	n/a	98.65	89,056	221,630	2.49	296	141/158

*M*_*n*_ number averaged molecular weight, *M*_*w*_ average molecular weight, *T*_*d*_ Degradation temperature, *T*_*m*_ Melting temperature, *n/a* not applicable

Table 1 shows the number-average molecular weight (*M*_n_) and the weight-average molecular weight (*M*_w_) along with the dispersity (*Đ*, *M*_w_/*M*_n_; a measure of the distribution of molecular mass in the polymer sample) of our PHA extracted from *C. necator* grown on CPH and compared with commercial PHBV. The *M*_n_ of the extracted PHA was around 6 × 10^5^ Da with a narrow distribution, indicating the production of PHA with a high molecular weight, higher than the commercial PHBV (CAS 80181-31-3) but comparable to the values obtained to those of heterotrophic enrichments of *Comamonas*, *Brachymonas* and *Acinetobacter* [61] and pure cultures of *Methylocystis parvus* OBBP [62].

**Fig. 3:**
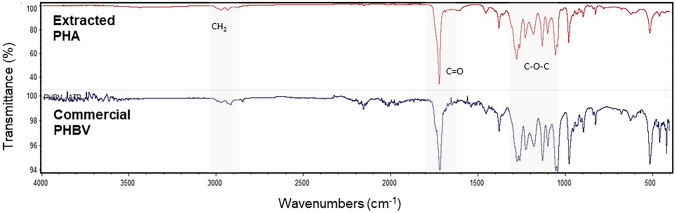
FT-IR spectra of PHA samples. Extracted PHA (red) and commercial PHBV (dark blue). Ranges highlighted in grey denote relevant bands (Color figure online)

Infrared spectroscopy analysis of PHA obtained from CPH along with spectra from commercial PHBV are shown in Fig. 3. All spectra showed typical functional group bands of PHA, such as the 2922–2929 cm^−1^ stretches that are the combination of CH_2_ asymmetric and symmetric stretching modes of the alkyl group. The observed 1724 and 1631 cm^−1^ bands is attributed to the C=O stretching that corresponds to the ester group present in the molecular chain of highly ordered crystalline structure, which was also reported by Vega-Castro et al. [58]. Representative bands of the C–O–C groups were also observed in the spectral region between 1050 and 1300 cm^−1^ (1184, 1230, and 1279 cm^−1^). Overall, the PHA produced from CPH using *C. necator* enabled the production of biopolymers containing the characteristic functional groups of commercial PHBV, thus, providing further evidence of the molecular composition of our copolymer. It has previously been reported that increased molar ratio (mol%) of 3HB:3HV in PHBV enhanced the polymer elongation compared to PHB alone but reduced the melting temperature [63]. Thus, the controlled 3HV mol% in PHBV polymer allows us to fine-tune its material properties, useful for a wider range of diverse applications.

### Melt Blending Experiments

PHA, such as the PHBV copolymer produced in this work, are regarded as biodegradable biopolymers but their potential applications are hampered due to brittleness arising from the formation of spherulites (i.e. crystallinity) [64]. The blending of PHA with a more flexible biodegradable polymer, e.g. polycaprolactone (PCL), offers a great opportunity to alter the thermal and mechanical properties to broaden the range of potential applications. PCL has a relatively low rate of hydrolytic degradation, which is known to speed up in the presence of other, more rapidly degrading polyesters, which produce acidic by-products during degradation. The presence of PCL will create a crystalline matrix, which is expected to slow down the rate of degradation of the PHBV polyester. Therefore, fully biodegradable PHBV blends were prepared with PCL as a plasticizer. The effects of mixing PCL on the chemical, thermal, morphological and mechanical properties of binary blends were investigated. We prepared blends of PHBV/PCL by melting the mixture with compositions of 70/30, 50/50, and 30/70 (% w/w) and studied their properties. Figure S2 shows the FT-IR spectra of the three binary blends alongside PHBV and PCL alone.

**Fig. 4:**
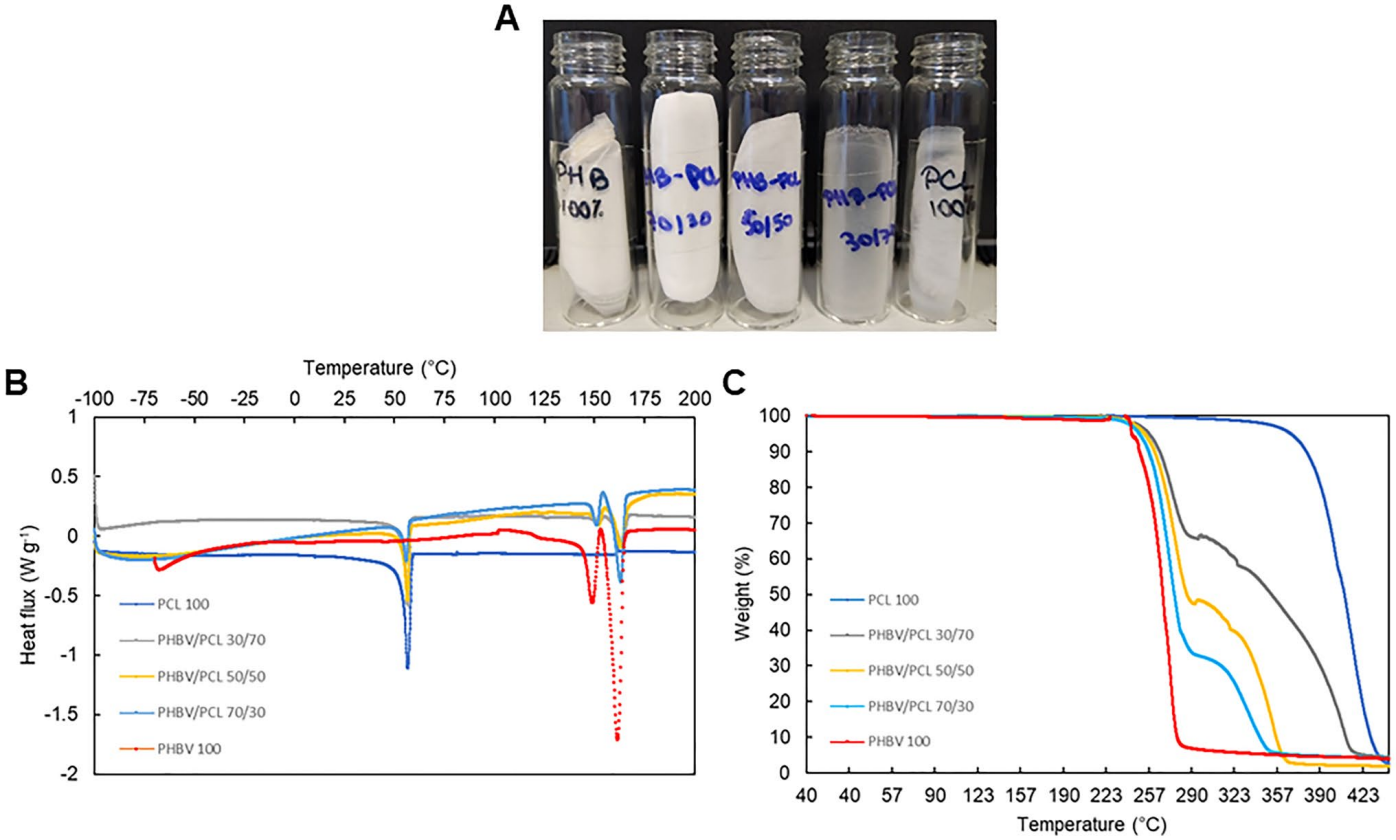
Aspect and thermal characterisation of the binary biopolymer PHBV/PCL blends. **A** Blended films and; **B** DSC and **C** TGA thermograms of the PHBV/PCL binary blends alongside HBV and PCL homopolymer samples

Where PHBV was used, the FTI-IR spectrum showed typical functional group band characteristics of PHA as discussed in “PHA production” section. Blends with an increasing proportion of PCL showed typical functional group band characteristics of PCL whereby the absorption band at 2940 cm^−1^ is assigned to the hydroxyl groups asymmetric stretching. The band at 2860 cm^−1^ is assigned to the hydroxyl groups symmetric stretching. Hence, FTIR showed the expected trends, and confirmed, as expected, that no reactive blending is occurring. Figure 4A shows the aspect of the obtained PHBV/PCL binary blends whereby the white-opaque colour denotes the presence of PHBV whereas the more translucent appearance donates the presence of PCL. Figure 4B depicts the DSC thermograms of the PHBV/PCL binary blends as well as the thermograms of the PHBV and PCL homopolymers. The melting point of the PCL was 55.6 ℃ whereas PHBV showed two melting points (143.4 ℃ and 158.3 ℃) due to cold crystallization peaks, respectively [65]. PHBV/PCL binary blends showed three melting temperatures. The presence of different melting temperatures, as evidenced in the thermograms, is related to the PCL and PHBV melting points. Figure 4B shows the TGA profiles of PHBV/PCL binary blends alongside PHBV and PCL homopolymers. As can be observed, the degradation temperature (T_d_) of the binary PHBV/PCL blends are within the values of 100% PHBV (267 ℃) and 100% PCL (424 ℃). An increase in PCL proportion increases T_d_. In addition, binary PHBV/PLC blends show two T_d_ peaks because of their composition. The blend with the lowest composition of PCL (30%, w/w) shows a 340 ℃ whereas the blend with the highest PCL composition (70%, w/w) exhibits a higher T_d_ at 380 ℃.

**Fig. 5:**
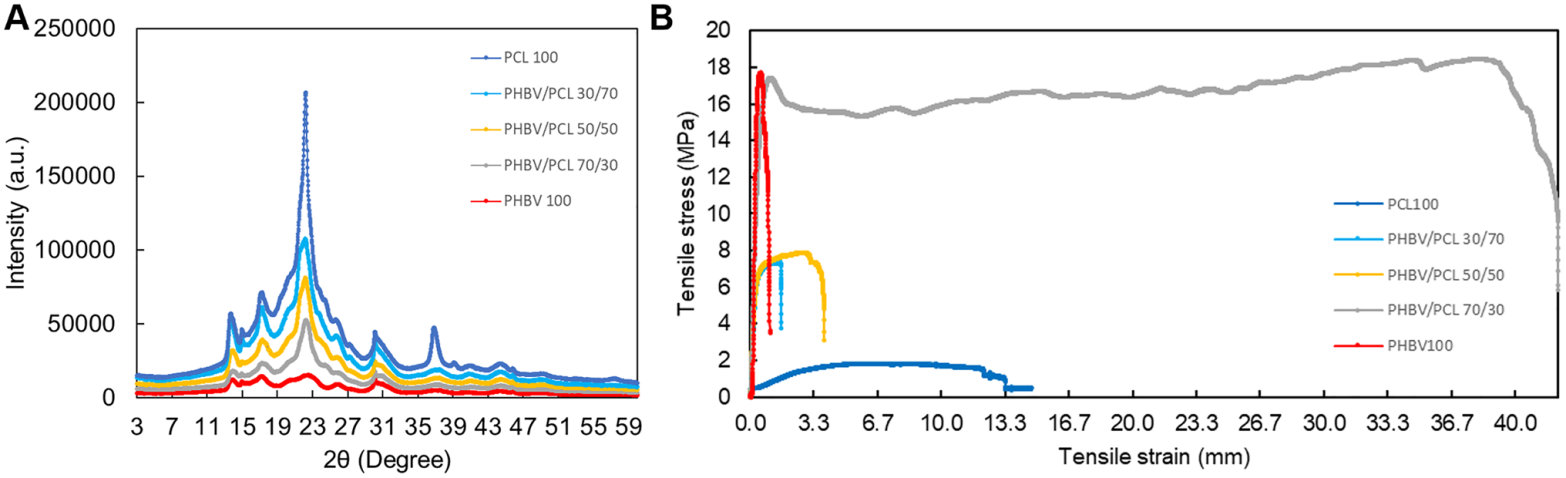
Characterisation of PHBV/PCL binary biopolymer blends using **A** X‐ray diffraction (XRD) and **B** tensile testing

X‐ray diffraction of biopolymer blends is shown in Fig. 5A. The peaks observed in XRD are around: (020) at 2θ = 13°, (110) at 2θ = 17°, (101) at 2θ = 22°, (111) at 2θ = 22°, (121) 2θ = 25° and (040) at 2θ = 31°. From Fig. 5A, the peaks (020) at 2θ = 13° and (110) at 2θ = 17° indicate orthorhombic unit cell individually, which are the most two intense and scattering peaks. Two relatively weaker peaks are observed at (101) at 2θ = 15° and (111) at 2θ = 25°, which correspond to α‐PHB crystal. The other two minor peaks observed at (121) 2θ = 25° and (040) at 2θ = 27° indicate the partial crystalline nature of PHB. The diffraction pattern and characteristic peaks of PHBV from this work are very similar to PHB obtained from *C necator* reported elsewhere [66]. The characteristic peaks of the PCL-containing blends with three different loadings were observed at 2θ = 21.3°, 22.4° and 23.7°, which are correspondent to the (110), (111), and (200) crystal faces, respectively [67]. The morphology analysis demonstrated that the blending did not develop new co-crystallisation when comparing to blends with 100% PHBV and 100% PCL.

The stress–strain curves, tensile strength, and elongation at break of PHBV/PCL binary blends with various PCL content are shown in Fig. 5B. The toughness of PHBV/PCL binary blends increases sharply with a higher content of PCL. PHBV blend breaks at least at 1% of strain stress whereas PCL breaks at 10% of strain stress. The 50/50 and 70/30 PHBV/PCL blends break at 2.8% and 1.1%, respectively. When the PCL content increases from 0 to 70 wt%, the elongation at the break of the PHBV/PCL blend improved by 28%. These results show that the incorporation of PCL in the PHBV blend can enhance the toughness of the binary blends. To obtain biopolymer blends of similar mechanical properties to some common plastics such as PET/PLA blends [68] and LLDPE/MMT fibres [69].

Table 2 summarises the crystallinity, mechanical and thermal properties of PHBV/PCL binary biopolymer blends.

**Table 2:** Physical, mechanical and thermal properties of PHBV/PCL binary biopolymer blends

	Crystallinity	Young modulus	Force at break	Elongation	T_d_	T_m_
	(%)	Mpa	Mpa	(%)	(°C)	(°C)
PHBV 100	59.5	12.3	3.5	0.68	267	150–159
PHBV/PCL 70/30	46.5	6.5	3.7	1.1	340	134–158
PHBV/PCL 50/50	47.8	5.1	3.1	2.5	360	55–134–158
PHBV/PCL 30/70	72.8	5.3	5.9	28.1	380	55–134–158
PCL 100	62.1	0.12	1.5	9.8	424	55

*T*_*d*_ Degradation temperature, *T*_*m*_ Melting temperature

## Conclusions

In this work, we demonstrate, for the first time, that the biodegradable biopolymer poly(3-hydroxybutyrate-*co*-3-hydroxyvalerate) (PHBV) can be produced from cassava peel waste via an integrated pre-treatment and fermentation process using *Cupriavidus necator,* followed by alkaline digestion. The processing conditions employed in this study allowed for PHBV production at a controlled molar ratio of 2:1 (3HB:3HV), which provides improved elongation quality compared to biotechnologies for producing PHB only, and useful for divergent material usages. Fully biodegradable PHBV blends were prepared with PCL to manipulate the properties. The chemical, thermal, morphological and mechanical properties of binary blends could be fine-tuned by altering the PCL loading, making less stiff films. Blends with PHBV present as the major component were brittle, whereas blends containing high (70 wt%) PCL contents showed an excellent balance between stiffness and plasticity. Furthermore, the presence of PCL produced plastic blends with higher degradation temperatures, thus providing enhanced stability properties. The significance of this work is two-fold: (i) provision of a potential solution to waste management that can benefit cassava processing industries (or other crop processing industries) and (ii) development of plastic materials that can be applied for example, to packaging and biomedical engineering hence, offering environmental and healthcare benefits. We will use these findings to investigate more complex ternary blends, using biodegradable additives to further control the final properties to broaden the scope of PHBV use.

### Supplementary Information

Below is the link to the electronic supplementary material.

Supplementary file1 (DOCX 107 KB)
