# Supplementary material for: Valorising Cassava Peel Waste Into Plasticized Polyhydroxyalkanoates Blended with Polycaprolactone with Controllable Thermal and Mechanical Properties
Source: J Polym Environ. 2024 Jan 27;32(8):3503–15. doi: 10.1007/s10924-023-03167-4 (PMC11330390; doi:10.1007/s10924-023-03167-4)
Supplement: Supplementary file 1 — Supplementary file1 (DOCX 107 KB) [file 10924_2023_3167_MOESM1_ESM.docx]

**Valorising cassava peel waste into plasticized polyhydroxyalkanoates blended with** **polycaprolactone with controllable thermal and mechanical properties**

Emma Martinaud^1,2,3,†^, Carmen Hierro-Iglesias^2,†^, Bawan Hadad^3^, Rob Evans^3^, Jakub Sacharczuk^3^, Daniel Lester^4^, Matthew J Derry^3^, Paul D Topham^3^, Alfred Fernandez-Castane^2,3,*^

**Supplementary materials:**

As depicted in Figure S1A, a maximum OD_600_ of 12, equivalent to 3.5 g/L of biomass, was achieved after 59 h of culture, although sugars were still available in the culture (Figure S1B). Specifically, 7.7 g of glucose and 13.9 g/L of TRS remained in the culture at the end of the fermentation. This indicates that 12.3 g/L of glucose were consumed. The cessasion of growth before the complete consumption of sugars might suggest that *C. necator* utilised all the essential nutrients available in the media, or potential inhibitor byproducts were accumulated in the culture. Regarding PHA production, concentration increased up to 28.6 % (g_PHA_/g_DCW_) after 48h of culture, corresponding to 1 g/L of PHA.

The process parameters specific growth rate (µ), biomass yield coefficient (Y_x/s_), product yield coefficient (Y_p/s_), and volumetric productivity of PHA (Y_p_) are presented in Table S1.


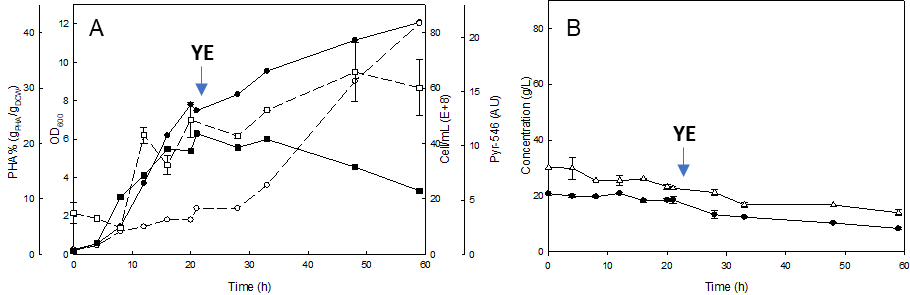


**Figure S1.** Fermentation profile showing (—●—) OD_600_; (--**○**--) cell/mL; (—■—) fluorescence Pyr-546 (pyrromethene 546)-stained cells; (--□--) PHA (polyhydroxyalkanoates) %; (—▲—) glucose (g/L) and (--Δ--) TRS (total reducing sugars) (g/L). AU: arbitrary units; (n=3).

**Table S1.** Process productivity parameters.

| µ_batch_ (h^-1^) | Y_X/S_ (g_biomass_/g_substrate_) | Y_P/S_ (g_PHA_/g_substrate_) | Y_P_ (g_PHA_/Lh) |
| --- | --- | --- | --- |
| 0.23 | 0.27 | 0.034 | 0.017 |

**
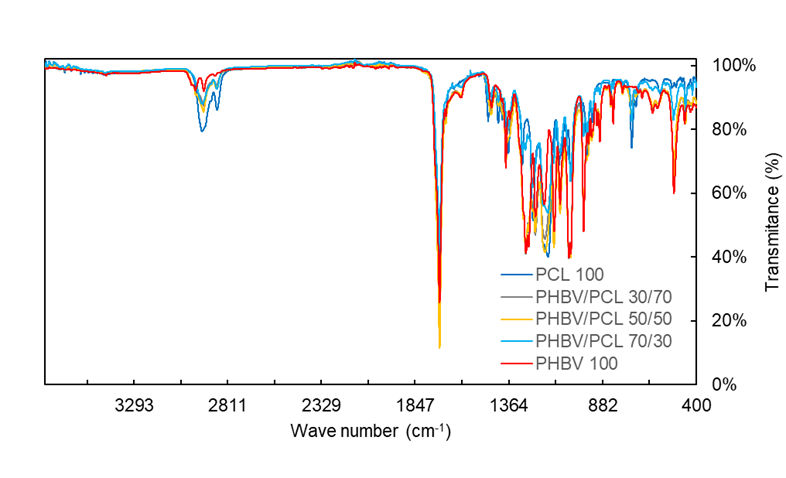
**

**Figure S1.** FT-IR spectra of binary PHBV/PCL blends.
